# (Pro)renin Receptor Inhibition Reduces Plasma Cholesterol and Triglycerides but Does Not Attenuate Atherosclerosis in Atherosclerotic Mice

**DOI:** 10.3389/fcvm.2021.725203

**Published:** 2021-12-24

**Authors:** Dien Ye, Xiaofei Yang, Liwei Ren, Hong S. Lu, Yuan Sun, Hui Lin, Lunbo Tan, Na Wang, Genevieve Nguyen, Michael Bader, Adam E. Mullick, A. H. Jan Danser, Alan Daugherty, Yizhou Jiang, Yidan Sun, Furong Li, Xifeng Lu

**Affiliations:** ^1^Department of Pharmacology, College of Pharmacy, Shenzhen Technology University, Shenzhen, China; ^2^Saha Cardiovascular Research Center and Department of Physiology, University of Kentucky, Lexington, KY, United States; ^3^Division of Pharmacology and Vascular Medicine, Department of Internal Medicine, Erasmus Medical Center, Rotterdam University, Rotterdam, Netherlands; ^4^Translational Medicine Collaborative Innovation Center, The Second Clinical Medical College (Shenzhen People's Hospital) of Jinan University, Shenzhen, China; ^5^Institut National de la Santé et de la Recherche Médicale (INSERM) and Collège de France Early Development and Pathologies Center for Interdisciplinary Research in Biology and Experimental Medicine Unit, Paris, France; ^6^Max-Delbrück Center for Molecular Medicine (MDC), Berlin, Germany; ^7^Institute for Biology, University of Lübeck, Lübeck, Germany; ^8^Charité University Medicine, Berlin, Germany; ^9^German Center for Cardiovascular Research (DZHK), Partner Site Berlin, Berlin, Germany; ^10^Ionis Pharmaceuticals, Inc, Carlsbad, CA, United States; ^11^Institute for Advanced Study, Shenzhen University, Shenzhen, China; ^12^Department of Physiology, Shenzhen University Health Science Center, Shenzhen University, Shenzhen, China

**Keywords:** macrophage, cholesterol, V-ATPase = vacuolar H+-adenosine triphosphatase, renin- angiotensin system, (Pro)renin receptor (PRR)

## Abstract

**Objective:** Elevated plasma cholesterol concentrations contributes to ischemic cardiovascular diseases. Recently, we showed that inhibiting hepatic (pro)renin receptor [(P)RR] attenuated diet-induced hypercholesterolemia and hypertriglyceridemia in low-density lipoprotein receptor (LDLR) deficient mice. The purpose of this study was to determine whether inhibiting hepatic (P)RR could attenuate atherosclerosis.

**Approach and Results:** Eight-week-old male LDLR^−/−^ mice were injected with either saline or N-acetylgalactosamine-modified antisense oligonucleotides (G-ASOs) primarily targeting hepatic (P)RR and were fed a western-type diet (WTD) for 16 weeks. (P)RR G-ASOs markedly reduced plasma cholesterol concentrations from 2,211 ± 146 to 1,128 ± 121 mg/dL. Fast protein liquid chromatography (FPLC) analyses revealed that cholesterol in very low-density lipoprotein (VLDL) and intermediate density lipoprotein (IDL)/LDL fraction were potently reduced by (P)RR G-ASOs. Moreover, (P)RR G-ASOs reduced plasma triglyceride concentrations by more than 80%. Strikingly, despite marked reduction in plasma lipid concentrations, atherosclerosis was not reduced but rather increased in these mice. Further testing in ApoE^−/−^ mice confirmed that (P)RR G-ASOs reduced plasma lipid concentrations but not atherosclerosis. Transcriptomic analysis of the aortas revealed that (P)RR G-ASOs induced the expression of the genes involved in immune responses and inflammation. Further investigation revealed that (P)RR G-ASOs also inhibited (P)RR in macrophages and in enhanced inflammatory responses to exogenous stimuli. Moreover, deleting the (P)RR in macrophages resulted in accelerated atherosclerosis in WTD fed ApoE^−/−^ mice.

**Conclusion:** (P)RR G-ASOs reduced the plasma lipids in atherosclerotic mice due to hepatic (P)RR deficiency. However, augmented pro-inflammatory responses in macrophages due to (P)RR downregulation counteracted the beneficial effects of lowered plasma lipid concentrations on atherosclerosis. Our study demonstrated that hepatic (P)RR and macrophage (P)RR played a counteracting role in atherosclerosis.

## Introduction

Atherosclerosis is a major cause of morbidity and mortality. Increased concentrations of plasma cholesterol and triglycerides, elevated blood pressure, and impaired blood glucose metabolism are the major risk factors for developing atherosclerosis and ischemic cardiovascular diseases (CVD). The (pro)renin receptor [(P)RR] can bind both renin and prorenin, and activate intracellular signaling cascades, including extracellular signal-regulated kinase 1/2 and phosphatidylinositol 3-kinase /Akt (1). Upon binding, the (P)RR activates prorenin in a non-proteolytic manner, leading to a renin-angiotensin system (RAS) activation. However, the interaction of (P)RR with renin/prorenin at supraphysiological concentrations questioned the physiological relevance of RAS. Indeed, recent studies show that the (P)RR is an accessory protein of the vacuolar H^+^-ATPase (V-ATPase) and is also indispensable for V-ATPase integrity and functions (2–5). We recently identified that the (P)RR played an RAS-independent role in regulating lipoprotein and lipid metabolism (6, 7). Suppressing the (P)RR in hepatocytes reduced the protein abundance of the low-density lipoprotein receptor (LDLR), which is the major receptor for low-density lipoprotein (LDL), thus reducing cellular LDL uptake (6). Inhibiting the hepatic (P)RR on one hand impairs plasma LDL clearance as a consequence of decreased hepatic LDLR abundance, but, on the other hand, it also reduces hepatic very low density lipoprotein (VLDL) secretion, resulting in a diet-dependent phenotype in plasma cholesterol (7). However, when LDLR functions are impaired, hepatic (P)RR inhibition reduces plasma cholesterol and triglycerides regardless of the diet being fed. We, thus, hypothesized that (P)RR inhibition would be an effective way to lower plasma cholesterol and triglycerides concentrations and to reduce the risk for atherosclerosis in familial hypercholesterolemia patients, whose LDLR activity is reduced or diminished, and whose responses to statin is less pronounced than in normal patients (8).

## Materials and Methods

The data that support the findings reported in this manuscript are available from the corresponding authors upon reasonable request.

### Animal Experiments

Low-density lipoprotein receptor deficient (LDLR^−/−^), ApoE^−/−^, and Lyz2-Cre mice were purchased from the Model Animal Research Center of Nanjing University (Nanjing, China), and mice carrying floxed (P)RR allele were kindly provided for by Prof. Michael Bader and Prof. Genevieve Nguyen (9). Mice were housed on a 10-h light/14-h dark cycle. Eight-week-old male LDLR^−/−^ and ApoE^−/−^ mice were subcutaneously injected on a weekly basis with either saline or N-acetylgalactosamine (GalNAc)-modified antisense oligonucleotides, therefore targeting the (P)RR [(P)RR G-ASOs]. Only male mice were studied because our previous study also focused on male mice. Also, the estrus cycle in female mice may affect atherosclerosis and other parameters (10). ASOs were synthesized as described before (7, 11, 12). G-(P)RR ASOs were injected subcutaneously at 3.0 mg/kg/week at the first 4 weeks and were then reduced to 1.5 mg/kg/week. Mice were fed a western-type diet (WTD, 42% kcal/kcal fat, 0.2% wt/wt cholesterol, cat Nr. TD88137, Envigo) for 16 weeks. Blood samples were collected via submandibular bleeding after 6 h of fasting. Systolic blood pressure was measured on conscious mice with a computerized noninvasive tail-cuff system (Softron, BP-2010A, Japan). Blood pressure was measured weekly for 4 weeks, prior to the end of the study. The mean of five repeated measurements at the last week (16th week) is reported. To isolate peritoneal macrophages, C57BL/6J mice were first injected with saline or 3.0 mg/kg (P)RR G-ASOs, then 4 days later, mice were injected with 6% autoclaved starch broth into its intraperitoneal cavity. Three days after starch broth injection, peritoneal macrophages were isolated as described (13). To obtain macrophage (P)RR knockout mice on ApoE^−/−^ background, Lyz2-Cre and (P)RR flox mice were first crossed with ApoE^−/−^ mice, and the offspring were intercrossed to obtain Lyz2-Cre^+/0^ (P)RR^wt/Y^ApoE^−/−^ mice and Lyz2-Cre^+/0^ (P)RR^fl/Y^ApoE^−/−^ mice, which were further intercrossed to obtain macrophage (P)RR knockout mice. Genotyping primers were listed in Supplementary Table 1. These mice were fed with WTD for 12 weeks to assess the consequences on atherosclerosis. Experimental procedures were approved by the Animal Ethics Committee of Shenzhen Health Science Center (no. 2014-0140).

### Isolation of Mouse Aortas and en Face Analysis

Aortic segments between the ascending aorta and the iliac arteries were dissected and fixed with 4% paraformaldehyde for 24 h. After fixation, adventitial tissues were carefully removed, and the aortas were cut open. Isolated aortas were quantified with or without Oil Red O (ORO), as described in the AHA statement (14). En face aortas were imaged with a microscope (Nikon, SMZ1270, Japan), and lesion areas were measured and quantified using Image J. In addition to quantification of plaque sizes in the whole aorta, plaque sizes of the ascending aorta, arch, and from the aortic orifice of left subclavian artery to 3 mm below were also quantified, which was designated as aortic arch in the figures.

### Histology Analysis of Aortic Root

Mice hearts were removed and fixed with 4% paraformaldehyde for 24 h, embedded in OCT, and cryosectioned at 7 μm thickness. Aortic root sections were prepared as recommended, but with some modifications (14). In short, serial tissue sections were acquired from the initial appearance of the aortic valves. Three tissue sections were placed on a single slide, and in total, 45–48 slides were obtained. The slide showing the largest aortic valves were chosen for hematoxylin and eosin (H&E) and ORO staining. Stained slides were scanned using Cytation 5 Cell Imaging Multi-mode reader (Biotek, US). Lesion areas of the aortic root were measured using ORO staining for the three sections on the same slide with Image J, and mean lesion size was reported.

### Biochemical Measurements

The total cholesterol of plasma and triglycerides concentration were measured by commercial kit (Wako, Japan) following the manufacturer's protocol. Plasma renin concentrations were measured by enzyme-kinetic assay in the presence of excess sheep angiotensinogen as described previously (15). The plasma concentrations of apolipoprotein B (ApoB) were determined by ELISA kit (Signalway Antibody, EK0320, US). Fractionation of plasma was described earlier (7), and cholesterol and triglycerides concentrations in each fraction were determined by commercial kit (Wako).

### RNA Isolation, Quantitative PCR, and RNA Sequencing

Total RNA was extracted using Direct-zolTM RNA MiniPrep kit (ZYMO Research). One microgram of total RNA was reverse-transcribed with Prime ScriptTM RT Master Mix (TaKaRa, Japan). SYBR Green real-time quantitative PCR assays were performed on a qTOWER apparatus (Analytic Jena, Germany) using SYBR® Premix Ex TaqTM II kit (TaKaRa). Primers used in the study were listed in Supplementary Table I. Total RNAs extracted from aortic arch region were used to construct RNA sequencing libraries, which were sequenced on Illumina HiSeq X10 platform. DESeq2 was used to identified differently expressed genes (DEGs). Gene ontology (GO) and KEGG enrichment analysis were performed using clusterProfiler. Gene set variation analysis (GSVA) was performed as described previously (16), using described curated datasets (Supplementary Table II) from the literature (17, 18).

### Cellular Experiments

RAW264.7 cells were maintained with DMEM high glucose medium supplemented with 10% fetal bovine serum. To inhibit (P)RR expression, 0.1 mg/ml final concentrations of (P)RR G-ASOs were incubated with cells. Twenty-four hours later, cells were incubated, with or without 100 ng/ml lipopolysaccharide (LPS, *In vivo*Gen), for 4 h to measuring gene abundance, and 12 h for measuring cytokine production, respectively. To stimulate cytokine release, cells were incubated with 10 μM nigericin for 30 min before collecting the cell culture medium. Concentrations of secreted cytokines in the cell culture medium were measured using commercial kits from Thermo Scientific (TNF-α: # 88-7324-88; IL-1b: # 88-7013-22; IL-6: # 88-7064-88; IL-10: 88-7105-88), following the manufacturer's protocol.

### Fast Protein Liquid Chromatography (FPLC) Analysis of Plasma Lipoproteins

Fast Protein Liquide Chromatography (FPLC) analysis was performed as described previously (7). In short, plasma samples from eight mice were pooled, and cleared by centrifugation and further filtered through a 0.22 μm filter. Two hundred fifty microliters of filtered plasma were loaded for FPLC analysis using Superous-6 Increase 10/300 GL column (GE) on an AKTA purifier (GE). Flow rate was set to 0.5 mL/min, and fractions between 10 and 16 mL were collected at an interval of 0.25 ml/fraction. Cholesterol and triglycerides concentrations in each fraction were measured.

### Statistics

All values are presented as mean ± SEM. The Kolmogorov-Smirnov test was performed to test normality. All samples passed normality test. Data were not tested for equal variance. Two-tailed Student *t*-test was performed when comparison was made between two groups: one-way ANOVA followed by the Bonferroni test was performed for comparison in case of >2 groups. *P* < 0.05 were considered significant. Statistical analysis was performed using Prism 9 (Graphpad Software).

## Results

### (P)RR G-ASOs Did Not Ameliorate but Increased Atherosclerosis in LDLR^–/–^ Mice

**Figure 1:**
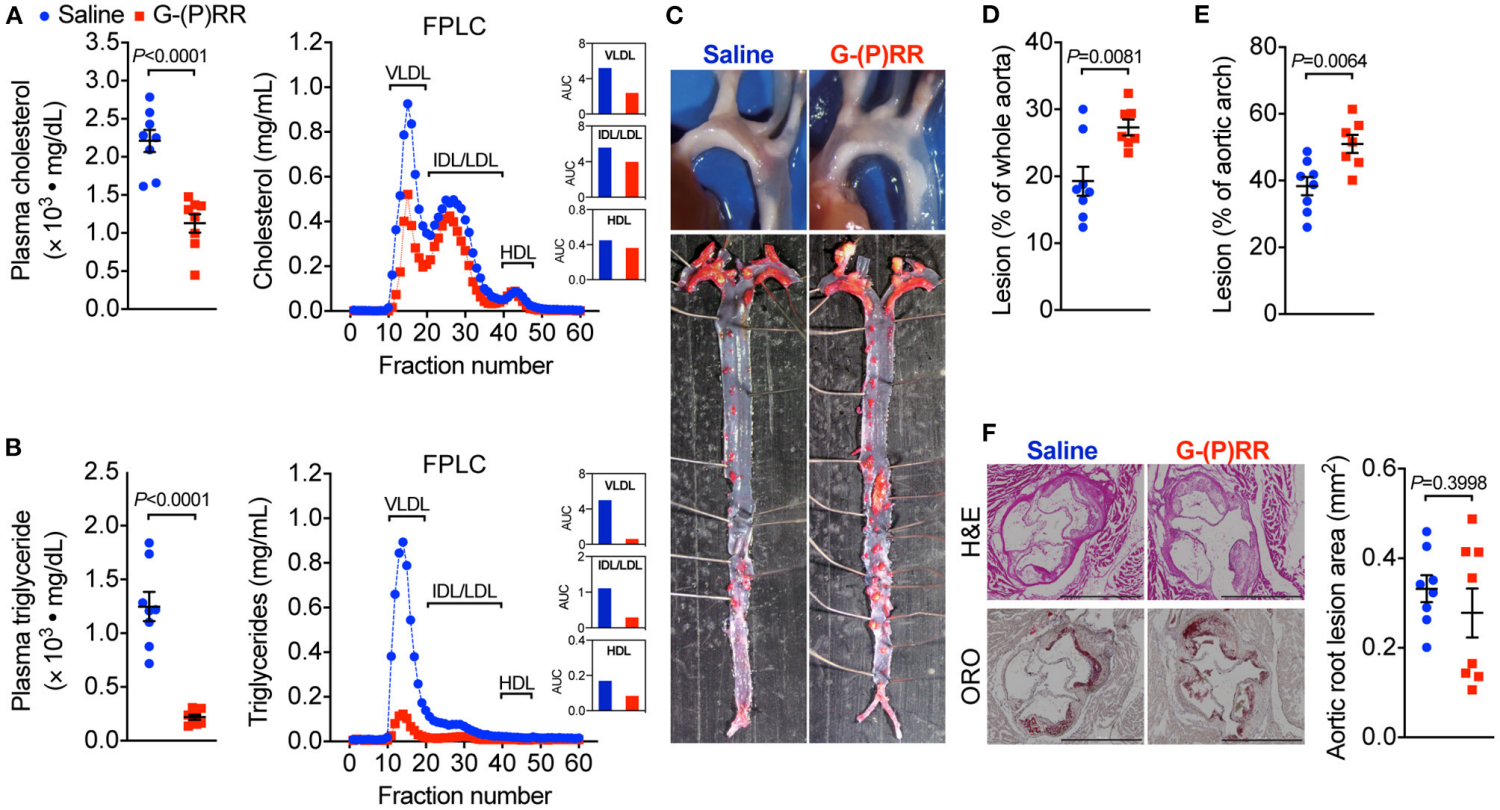
(Pro)renin receptor [(P)RR] N-acetylgalactosamine-modified antisense oligonucleotides (G-ASOs) reduced plasma cholesterol and triglyceride concentrations but not atherosclerosis in low-density lipoprotein receptor deficient (LDLR^−/−^) mice fed a western-type diet (WTD). Eight-week-old male LDLR^−/−^ mice were administered with saline (blue) or (P)RR G-ASOs (red) and fed a WTD for 16 weeks. *N* = 8/group. Total plasma cholesterol **(A)** and triglycerides concentrations **(B)**. Pooled plasma samples were resolved by FPLC for lipoprotein fractionation analysis, and cholesterol **(A)** and triglycerides content **(B)** in each fraction was determined. Representative images of the aorta arch and Oil Red O-stained whole aorta **(C)**. Quantification of lesion areas of the whole aorta **(D)** and aortic arch region **(E)**. Representative images of cross-sectioned aortic root stained with H&E and ORO and quantification of lesion areas of the aortic root **(F)**. Bar = 1,000 μm.

To evaluate whether hepatic (P)RR inhibition attenuates atherosclerosis, we administered (P)RR G-ASOs to LDLR^−/−^ mice. The efficacy and specificity of G-(P)RR ASOs in reducing hepatic (P)RR were demonstrated in our previous study (7). Inhibiting the (P)RR in hepatocytes reduced the plasma cholesterol concentrations by ~50% (2,211 ± 146 vs. 1128 ± 121 mg/dL), and FPLC analysis revealed that the cholesterol contents of VLDL and IDL/LDL fractions were the most reduced (Figure 1A). Plasma triglyceride concentrations were also significantly reduced, mainly by reducing VLDL and IDL/LDL-triglycerides (Figure 1B). Strikingly, lesion sizes in the aortic arch region and the entire aorta were increased rather than reduced by (P)RR inhibition (Figures 1C–E). However, lesions sizes in the aortic root, revealed by H&E and ORO staining, were unaltered by (P)RR inhibition (Figure 1F). It is worthy to notice that lesions development in the aortic root and in the aortic region can exhibit considerable differences (14, 19). Overall, our results indicate that atherosclerosis was accelerated by (P)RR G-ASOs. A previous study reported that systolic blood pressure was elevated in adipose (P)RR knockout mice (20), suggesting that (P)RR inhibition may affect RAS activity and blood pressure. However, (P)RR inhibition did not alter systolic blood pressure or plasma renin concentrations in LDLR^−/−^ mice (Supplementary Figure I), ruling out altered RAS activity as the counteracting factor for the beneficial effects of lowered lipid concentrations.

### (P)RR G-ASOs Did Not Ameliorate Atherosclerosis in ApoE^–/–^ Mice

**Figure 2:**
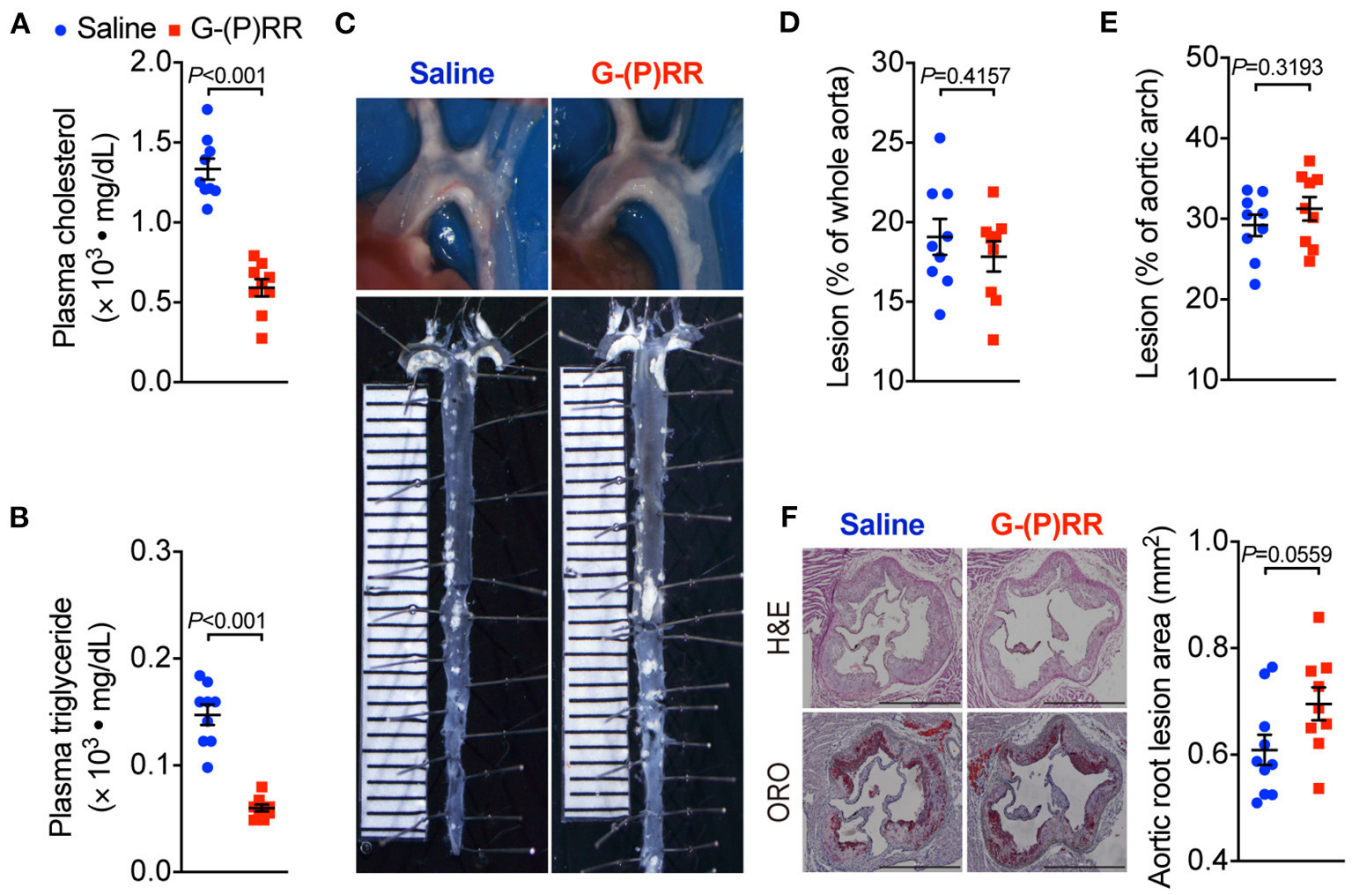
(P)RR G-ASOs reduced plasma lipid concentrations, but not atherosclerosis in ApoE^−/−^ mice. Eight-week-old ApoE^−/−^ mice were administered with either saline (blue) or (P)RR G-ASO (red) and fed a WTD for 16 weeks. N=9 per group. Plasma cholesterol **(A)** and triglycerides concentrations **(B)**. Representative images of aortic arch and en face whole aorta **(C)**. Quantification of lesions in whole aorta **(D)** or aortic arch region **(E)**. Representative images showing H&E and ORO-stained sectioned aortic root and quantification of lesion size in aortic root **(F)**. Bar = 1,000 μm.

We further tested the effect of (P)RR inhibition on atherosclerosis in another atherosclerotic mice model, namely ApoE^−/−^ mice. We found that (P)RR G-ASOs also effectively reduced both plasma cholesterol and triglyceride concentrations in ApoE^−/−^ mice (Figures 2A,B; Supplementary Figure II), into a similar extent as observed in LDLR^−/−^ mice. Since plasma lipid-lowering effects were observed in both LDLR^−/−^ and ApoE^−/−^ mice, while (P)RR inhibition reduces hepatic LDLR abundance, the results therefore indicate that LDLR and ApoE were not required for the lipid-lowering effects of (P)RR inhibition. However, despite the marked reduction in plasma lipids concentrations, lesion size in the whole aorta and aortic arch region has remained unaltered by (P)RR inhibition (Figures 2C–E). Moreover, lesion sizes of the aortic root, revealed by H&E and ORO staining, were also unaltered by (P)RR G-ASOs (Figure 2F). As a whole, it is clear that (P)RR G-ASOs were unable to attenuate atherosclerosis in ApoE^−/−^ mice.

### (P)RR G-ASOs Promoted Immune Responses by Stimulating Macrophage Inflammatory Cytokines

**Figure 3:**
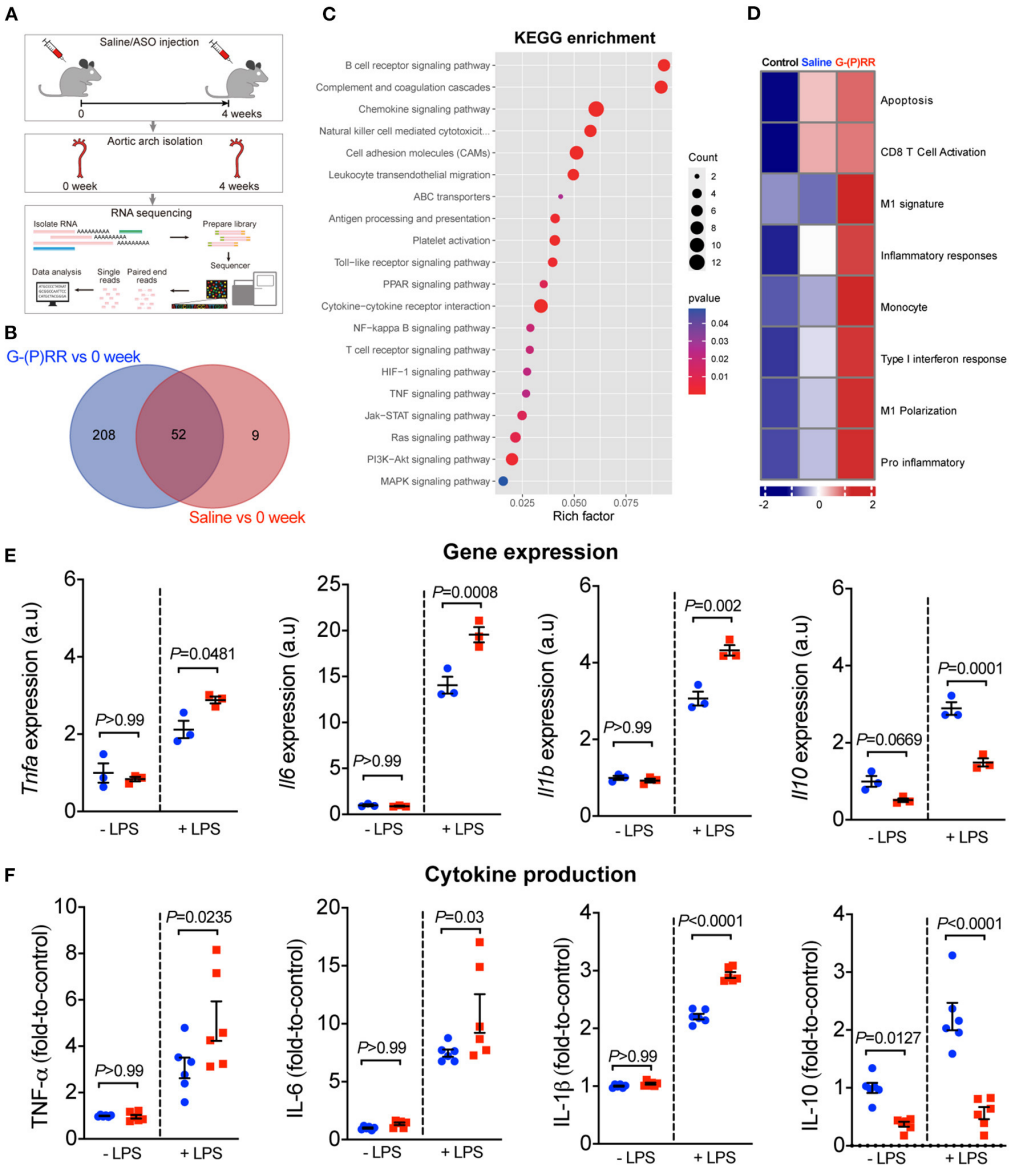
(P)RR G-ASOs promoted immune responses in aorta by augmenting macrophage inflammatory cytokine production. **(A–D)** Eight-week-old LDLR^−/−^ mice were administered with either saline or (P)RR G-ASOs and fed a WTD for 4 weeks, and aortas were isolated for transcriptomic analyses. Aortas from 8-week-old LDLR^−/−^ mice (0 week) served as control. Experimental procedure **(A)**. Venn graph showing overlapped and non-overlapped DEGs **(B)**. KEGG enrichment analysis **(C)** and GSVA analysis of curated gene sets **(D)**. **(E,F)** RAW264.7 cells were incubated with saline (blue) or (P)RR G-ASOs (red) and stimulated with or without LPS. Expression and production of cytokines were determined.

**Figure 4:**
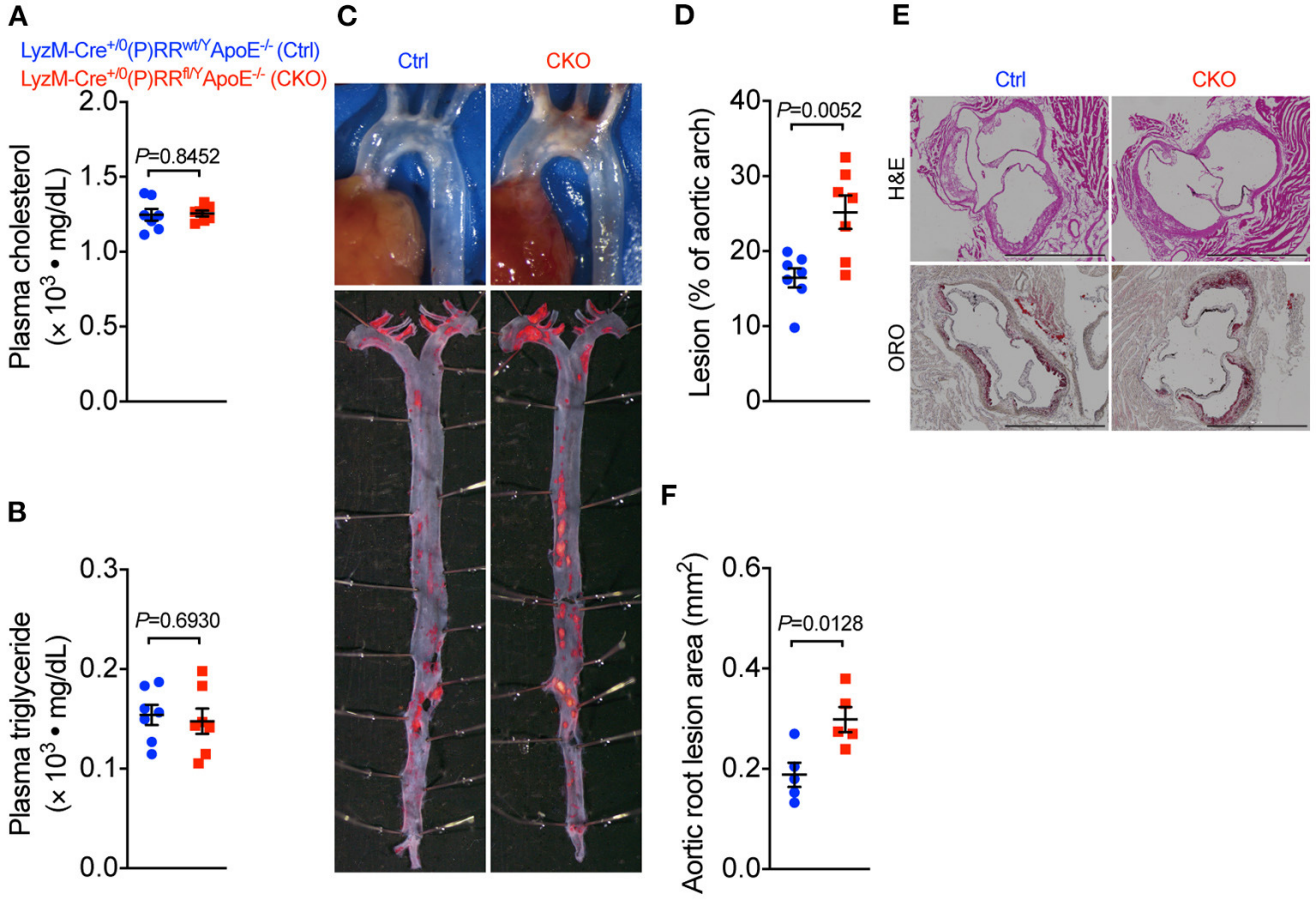
Deleting *(P)RR* in macrophages accelerated atherosclerosis in ApoE^−/−^ mice. Eight-week-old Lyz2-Cre^+/0^ (P)RR^wt/Y^ApoE^−/−^ mice (Ctrl) and Lyz2-Cre^+/0^ (P)RR^fl/Y^ApoE^−/−^ mice (CKO) were fed a WTD for 12 weeks. *N* = 7 per group. Plasma cholesterol and triglycerides concentrations **(A,B)**. Representative images showing aortic arch and ORO-stained whole aorta **(C)**. Quantification of lesion area in whole aorta **(D)** and aortic arch region **(E)**. Representative images showing H&E and ORO-stained sections of aortic root and quantification of lesion size in aortic root **(F)**. Bar = 1,000 μm.

Our observation that (P)RR G-ASOs markedly reduced plasma lipid concentrations, but not atherosclerosis in two different atherosclerotic models, suggested that (P)RR G-ASOs can promote other crucial atherogenic factor(s) to counteract the potential benefit of lowered plasma lipids. To clarify this, we mapped the transcriptomic changes in aortic arch region of saline and (P)RR G-ASOs administered LDLR^−/−^ mice that was fed with a WTD for 4 weeks (Figure 3A). Using aortic arch region of normal diet-fed 8-week-old LDLR^−/−^ mice as control, we identified 58 upregulated and 3 downregulated genes in saline administered LDLR^−/−^ mice, and the GO enrichment analysis of DEGs revealed that immune response-related biological processes were the most affected (Supplementary Table III; Supplementary Figure III). In comparison, (P)RR G-ASOs upregulated 256 genes and downregulated 4 genes, which are also enriched in immune- response-related biological processes (Supplementary Table IV; Supplementary Figure IV). We then compared the DEGs and found that 206 genes were specifically altered by (P)RR G-ASOs (Supplementary Table V; Figure 3B). GO enrichment analysis revealed that these genes were related to immune responses (Supplementary Figure V), while KEGG enrichment analysis revealed that inflammation related pathways, such as NF-κB signaling pathway, chemokine signaling pathway, and Toll-like receptor signaling pathway, were mostly affected (Figure 3C). GSVA analysis demonstrated that inflammatory gene set, which is type I interferon response gene set, and M1 macrophage signature gene set were activated (Figure 3D), suggesting enhanced inflammation in the aortic arch region of (P)RR G-ASOs administered LDLR^−/−^ mice. Despite the relatively high specificity of GalNAc-modified ASOs toward hepatocytes (21), macrophages could also be a target as they express asialoglycoprotein receptors which bind GalNAc (22). We therefore suspected that (P)RR G-ASOs may inhibit (P)RR expression in macrophages and consequently promotes inflammation, thereby counteracting the benefit of lowered plasma lipids concentrations. Indeed, isolated peritoneal macrophages from C57BL/6J mice administered (P)RR G-ASO for 1 week have showed marked reduction in (P)RR expression (Supplementary Figure VIA). Moreover, a successfully inhibited (P)RR expression in murine RAW264.7 (Supplementary Figure VIB), a widely used murine macrophage cell line, has enhanced LPS-stimulated expression of pro-inflammatory cytokines, including *Tnfa, Il6* and *Il1b* (Figure 3E). In RAW264.7 cells, inhibiting the (P)RR has reduced the abundance of *Il10*, which is an anti-inflammatory cytokine, done either with or without LPS stimulation (Figure 3E). In line with the gene expression findings, (P)RR inhibition in RAW264.7 increased TNF-α, IL-6 and IL-1β secretion, and decreased IL-10 secretion (Figure 3F; Supplemental Figure VII). To confirm the effect of macrophage (P)RR downregulation in atherosclerosis, we generated macrophage-specific (P)RR knockout mice on ApoE^−/−^ background (Supplementary Figure VIII). We found that deleting the (P)RR in macrophages did not affect plasma cholesterol and triglyceride concentrations (Figures 4A,B), further confirming that lowered plasma lipid concentrations were due to hepatic (P)RR deficiency. But, macrophage (P)RR-deleted mice did show accelerated atherosclerosis development (Figures 4C–F). Collectively, these findings suggested that suppressed (P)RR expression in macrophages has enhanced inflammatory responses in lesions, counteracting the benefits of lowered plasma lipid concentrations by hepatic (P)RR inhibition.

## Discussion

Increased plasma cholesterol concentrations are thought to initiate atherosclerosis by causing abnormal lipid deposition in the artery wall (23). Reducing plasma cholesterol concentrations, for instance, with statins, is an effective way to reduce CVD risk. However, in the current study, we demonstrated that a ~50% reduction in plasma cholesterol concentrations by hepatic (P)RR inhibition failed to attenuate atherosclerosis in either LDLR^−/−^ or ApoE^−/−^ mice. It is worthy to note that reduced plasma cholesterol concentrations were observed as early as 1 week after hepatic (P)RR inhibition (7). Thus, the reduction of plasma cholesterol concentrations was maintained throughout the experimental period. Moreover, IDL/LDL cholesterol, the most potent atherogenic cholesterol, was reduced by ~30% by inhibiting hepatic (P)RR inhibition. LDL particles are heterogenous in terms of size, density, and lipid compositions, and LDL particles with smaller size and higher density (more apolipoproteins and less lipids) have higher atherogenic ability (24, 25). Thus, it is possible that (P)RR inhibition alters the size and density of LDL. Indeed, we found that plasma ApoB concentrations were increased by inhibiting hepatic *(P)RR* (Supplementary Figure IX). Since sortilin-1 promotes ApoB degradation and the (P)RR inhibition reduces sortilin-1 abundance (6, 7, 26), increased ApoB is likely a result of reduced sortilin-1 abundance. This finding, together with reduced cholesterol content in the IDL/LDL fraction, suggested that the densities of LDL particles were likely increased by hepatic (P)RR inhibition. Since small dense LDL enters arterial intima more easily and is more prone to be oxidized (27, 28), it may also elicit immune responses smoothly. This may explain the overactivated immune responses in the aortas of (P)RR inhibited mice.

Activation of immune responses, characterized by infiltration of macrophages, mast cells, and T lymphocytes, is another hallmark in atherosclerosis development (29). The number of macrophages accumulating in aorta can increase up to 20-fold during atherogenesis (30). These macrophages can internalize accumulated oxidized LDL, leading to foam cell formation and the production of inflammatory cytokines, such as IL-1β. Oxidized LDL can also directly interact with Toll-like receptors to activate the expression of proinflammatory cytokines and chemokines (29), which leads to activation of both innate and adaptive immune responses. Unexpectedly, we found that inhibiting macrophagic (P)RR promoted inflammatory cytokine production in the presence of exogenous stimuli, providing another possibility of why (P)RR G-ASOs did not attenuate atherosclerosis although they did reduce plasma lipid concentrations. A recent study demonstrated that WTD feeding increased angiotensinogen, as well as the angiotensin type 1 receptor expression in peritoneal macrophages, while blocking the RAS with the angiotensin type 1 receptor antagonist, valsartan, reduced ox-LDL concentrations, and expression of *Il1b* and *Tnfa* in macrophages (31). This study also highlighted that RAS activation plays a role in inflammatory responses of macrophages. However, despite the debate on the role of (P)RR in RAS (32), inhibiting the (P)RR would inhibit rather than activate the RAS. Thus, (P)RR depletion is an unlikely cause of macrophage inflammatory responses via RAS activation. In fact, we found no effect on renin concentrations by (P)RR G-ASOs. Thus, the observed effect might be linked with its functions related to V-ATPase. V-ATPase is expressed at the plasma membrane and in lysosomes in macrophages (33). Inhibiting V-ATPase using bafilomycin induces TNF-α production in macrophages with and without LPS stimulation, and extends the duration of LPS-stimulated TNF-α production (33). Moreover, deleting macrophage *Atp6v0d2*, a subunit of the V-ATPase complex, augmented LPS-stimulated IL-1β and TNF production *in vivo* (34). Interestingly, this study also showed that LPS stimulation itself reduced *Atp6v0d2* expression, indicating that inhibition of V-ATPase is required for activating inflammatory responses in macrophages. V-ATPase also plays a role in cholesterol efflux in macrophages (35). Inhibiting V-ATPase using bafilomycin dose-dependently reduced ATP cassette, binding protein A1-mediated cholesterol efflux in RAW264.7 cells, inhibiting V-ATPase using bafilomycin dose-dependently reduced cholesterol efflux mediated by ATP cassette binding protein A1. In fact, our transcriptomic result shows that the expression of ATP-binding cassette transporters was altered by (P)RR G-ASOs. Thus, it is possible that (P)RR deficiency in macrophages augments inflammation and impairs cholesterol efflux by impairing V-ATPase activity.

In summary, we showed that (P)RR G-ASOs lowered the plasma lipid concentrations in WTD-fed LDLR^−/−^ and ApoE^−/−^ mice due to hepatocyte (P)RR inhibition. However, unexpectedly, downregulation of (P)RR in macrophages due to (P)RR G-ASOs promotes inflammatory cytokine production and suppressed anti-inflammatory cytokine production, thus counteracting the benefits of lowering plasma lipid concentrations. Overall, (P)RR G-ASOs did not attenuate atherosclerosis in WTD-fed LDLR^−/−^ and ApoE^−/−^ mice.

## Data Availability Statement

The datasets presented in this study can be found in online repositories. The names of the repository/repositories and accession number(s) can be found below: NCBI accession number: GSE167972.

## Ethics Statement

The animal study was reviewed and approved by Animal Ethic Committee of Shenzhen Health Science Center.

## Author Contributions

DY and XY: performed the experiments, analyzed the data, and wrote the draft manuscript. LR, YuS, NW, HL, and LT: assisted in acquiring and analyzing the data, and preparation of the manuscript. FL and XL designed the study and revised the manuscript. HSL: provided technical guidance over the execution of the study. HSL, GN, MB, AHJD, AD, AM, YJ, and YiS: discussed the data and revised the manuscript. All authors contributed to the article and approved the submitted version.

## Funding

The author XL is supported by National Natural Science Foundation of China (81870605), Shenzhen Municipal Science and Technology Innovation Council (JCYJ20190808170401660), and Shenzhen Key Laboratory of Metabolism and Cardiovascular Homeostasis (ZDSYS20190902092903237). YuS is supported by National Natural Science Foundation of China (81800383). FL is supported by National Natural Science Foundation of China (81670702), and Shenzhen Municipal Science and Technology Innovation Council (GJHZ20170310090257380). AHJD is supported by the Top Institute Pharma (T2-301). YiS is supported by Guangdong Basic and Applied Basic Research Foundation (2019A1515110993). YJ is supported by Shenzhen Municipal Science and Technology Innovation Council (Grant No. JCYJ20180305124812444).

## Conflict of Interest

AM is an employee and shareholder of Ionis Pharmaceuticals. The remaining authors declare that the research was conducted in the absence of any commercial or financial relationships that could be construed as a potential conflict of interest.

## Publisher's Note

All claims expressed in this article are solely those of the authors and do not necessarily represent those of their affiliated organizations, or those of the publisher, the editors and the reviewers. Any product that may be evaluated in this article, or claim that may be made by its manufacturer, is not guaranteed or endorsed by the publisher.

## Abbreviations

CVD: cardiovascular diseases
GalNAc: N-acetylgalactosamine
IDL: intermediate density lipoprotein
LDL: low-density lipoprotein
LDLR: low-density lipoprotein receptor
LPS: lipopolysaccaride
ox-LDL: oxidized LDL
(P)RR: (pro)renin receptor
RAS: renin-angiotensin system
TNF: tumor necrosis factor
V-ATPase: vacuolar H^+^-ATPase
VLDL: very low density lipoprotein
WTD: western type diet.
