# Supplementary material for: (Pro)renin Receptor Inhibition Reduces Plasma Cholesterol and Triglycerides but Does Not Attenuate Atherosclerosis in Atherosclerotic Mice
Source: Front Cardiovasc Med. 2021 Dec 24;8:725203. doi: 10.3389/fcvm.2021.725203 (PMC8739895; doi:10.3389/fcvm.2021.725203)
Supplement: Supplementary file 1 [file Data_Sheet_1.DOCX]

Supplementary Figures and Tables

**Supplementary Table I. Primers used.**

| Target | Forward | Reverse |
| --- | --- | --- |
| *(P)RR* | 5'-GGGTGGATAAACTGGCACTTC-3' | 5'-TGGAATTTGCAACGCTGTC-3' |
| *36b4* | 5'-ACTGGTCTAGGACCCGAGAAG-3' | 5'-CTCCCACCTTGTCTCCAGTC-3' |
| *Il1b* | 5'-AACTGTTCCTGAACTCAACTGT-3' | 5'-GAGATTTGAAGCTGGATGCTCT-3‘ |
| *Tnfa* | 5'-AAGCCTGTAGCCCACGTCGTA-3' | 5'-GGCACCACTAGTTGGTTGTCTTTG-3' |
| *Il6* | 5'-TAGTCCTTCCTACCCCAATTTCC-3' | 5'-TTGGTCCTTAGCCACTCCTTC-3' |
| *Il10* | 5'-GCTGCGGACTGCCTTCA-3' | 5'-TGCATTAAGGAGTCGGTTAGCA-3' |
| Lf | 5'-AGCACTCTCTTCCAGGTATGTTGTG-3' |  |
| Lr | 5'-CTGGATCCCGGAGCATGGGTAAAGG-3' |  |
| Er | 5'-GCCCCTCTCTTACAGTTCTATCAGT-3' |  |
| Cre | 5'-GCTGCCACGACCAAGTG-3' | 5'-TCGCCATCTTCCAGCAG-3' |


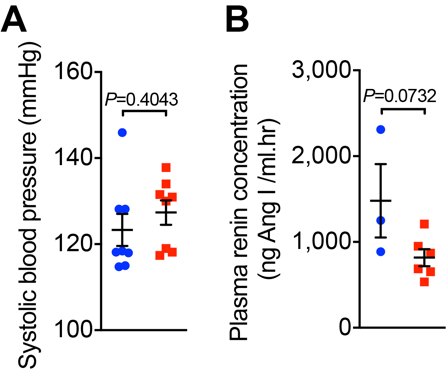


**Supplementary Figure I**. Systolic blood pressure (**A**) and plasma renin activity (**B**) of saline (blue) or (P)RR G-ASOs (red) treated LDLR^-/-^ mice fed with WTD for 16 weeks.


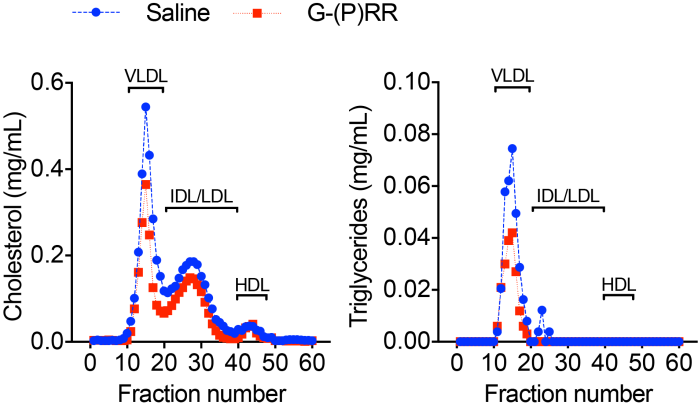


**Supplementary Figure II**. Pooled plasma samples from Saline (blue) or (P)RR G-ASOs (red) treated HFD-fed ApoE^-/-^ mice were resolved by FPLC for lipoprotein fractionation analysis, and cholesterol and triglycerides content in each fraction were determined.


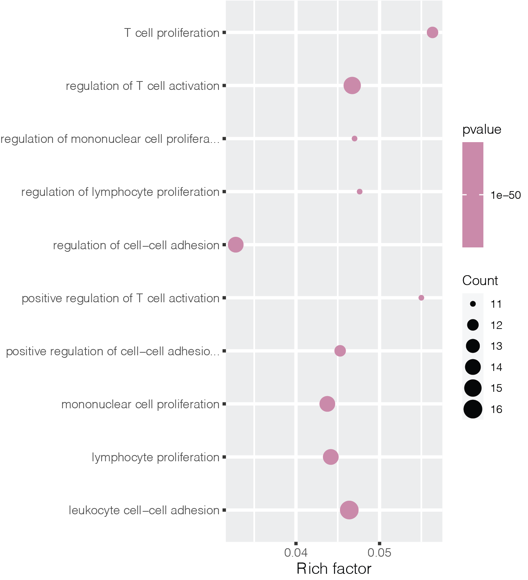


**Supplementary Figure III. GO enrichment analysis of biological processes.** DEGs (saline treated WTD-fed LDLR^-/-^ mice for 4 weeks vs 0-week control) were used.


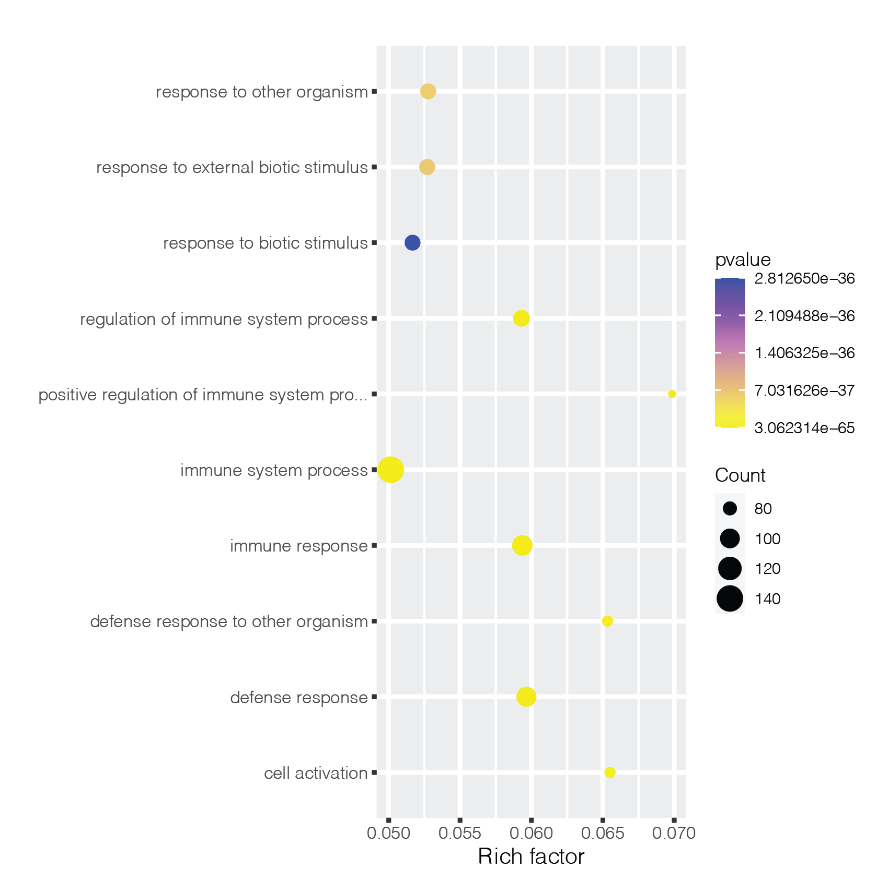


**Supplementary Figure IV. GO enrichment analysis of biological processes.** DEGs [(P)RR G-ASOs administered WTD-fed LDLR^-/-^ mice for 4 weeks vs 0-week control] were used.


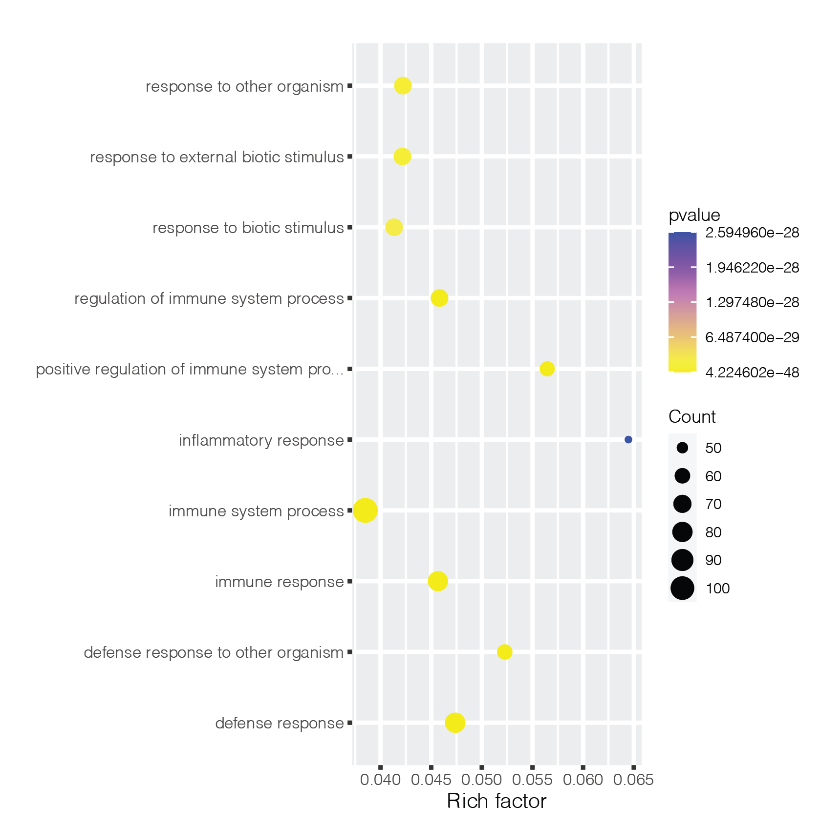


**Supplementary Figure V. GO enrichment analysis of biological processes.** Non-overlapped DEGs {[(P)RR G-ASOs 4 weeks vs 0 week] vs (saline 4 weeks vs 0 week)} were used.


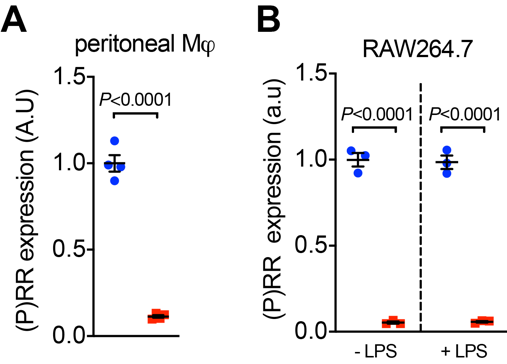


**Supplementary Figure VI**. (P)RR expression in ex vivo peritoneal macrophages (A) and RAW264.7 cells (B). Blue: saline; Red: (P)RR G-ASOs.


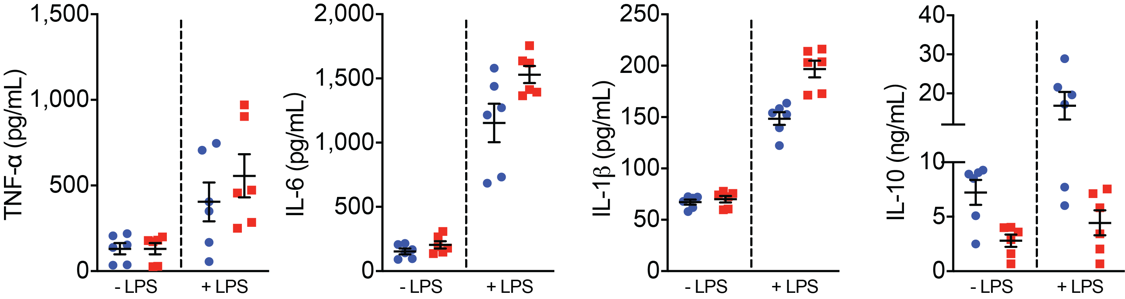


**Supplementary Figure VII**. RAW264.7 cells were incubated with saline (blue) or (P)RR G-ASOs (red), and stimulated with or without LPS. Concentrations of the indicated cytokines were determined.


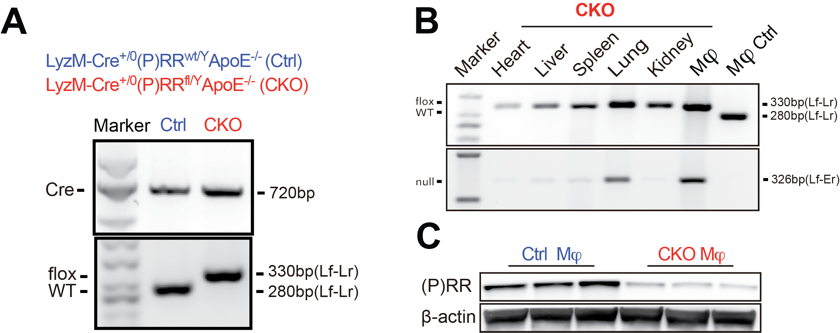


**Supplementary Figure VIII. Deleting (P)RR in macrophages in ApoE^-/-^ mice.** Genotyping of the mice (**A**). Deletion of (P)RR in macrophages was confirmed by PCR and Western blotting **(B&C**).


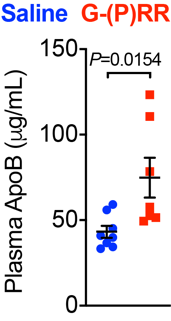


**Supplementary Figure IX.** Plasma ApoB concentrations of saline (blue) or (P)RR G-ASOs (red) administered LDLR^-/-^ mice fed a WTD for 16 weeks.
